# AI-Based Positioning with Input Parameter Optimization in Indoor VLC Environments

**DOI:** 10.3390/s22218125

**Published:** 2022-10-24

**Authors:** Sung-Hyun Oh, Jeong-Gon Kim

**Affiliations:** Department of Electronic Engineering, Korea Polytechnic University, Siheung-si 15297, Korea

**Keywords:** indoor positioning, localization, visible light communication (VLC), artificial Intelligence (AI), deep neural network (DNN), weighted k-nearest neighbor (WKNN)

## Abstract

Indoorlocation-based service (LBS) technology has been emerged as a major research topic in recent years. Positioning technology is essential for providing LBSs. The existing indoor positioning solutions generally use radio-frequency (RF)-based communication technologies such as Wi-Fi. However, RF-based communication technologies do not provide precise positioning owing to rapid changes in the received signal strength due to walls, obstacles, and people movement in indoor environments. Hence, this study adopts visible-light communication (VLC) for user positioning in an indoor environment. VLC is based on light-emitting diodes (LEDs) and its advantage includes high efficiency and long lifespan. In addition, this study uses a deep neural network (DNN) to improve the positioning accuracy and reduce the positioning processing time. The hyperparameters of the DNN model are optimized to improve the positioning performance. The trained DNN model is designed to yield the actual three-dimensional position of a user. The simulation results show that our optimized DNN model achieves a positioning error of 0.0898 m with a processing time of 0.5 ms, which means that the proposed method yields more precise positioning than the other methods.

## 1. Introduction

With the progress of the fourth industrial revolution, large and complex buildings are being built in downtown areas. The increasing demand for location-based services (LBS) is gradually extending from outdoors to indoors and indoor positioning systems (IPS) or indoor localization systems (ILS) are attracting scientific and enterprise interest since there is a huge market opportunity for applying indoor positioning, mapping, and navigation technologies [1]. Over the past few decades, highly reliable and accurate global positioning systems (GPS) have generally been used in positioning systems for outdoor environments [2]. However, when GPS is applied to an indoor environment, its positioning accuracy decreases significantly due to propagation loss caused by obstacles and walls [3]. Moreover, the current IPS applications still face several technical and non-technical challenges, such as the quality of positioning services, location privacy and the availability of indoor maps [4]. To solve this problem, studies are being conducted to determine the location of indoor users more precisely [5,6].

These studies are based on wireless communication technologies and positioning algorithms developed for indoor environments. Representative mobile communication technologies used for indoor positioning include Wi-Fi [7], Bluetooth [8], and ultra-wideband (UWB) [9]. These technologies are based on radio frequency (RF), and although their positioning accuracies are somewhat limited, each of these technologies offers distinct advantages [10]. Recently, visible-light-communication (VLC)-based positioning technology has been studied as a promising method to overcome the limitation of RF-based positioning technologies. VLC technology can use light-emitting diodes (LEDs) to realize data communication and the communication and lighting functions of the LEDs can be used simultaneously. In addition, LEDs are replacing the existing indoor lighting fixtures owing to their advantages such as economy and eco-friendliness and they have been established as one of the next-generation mobile communication technologies [11,12].

The existing indoor positioning algorithms can be divided into range-based algorithms and range-free algorithms [13,14]. Between these two types, range-based algorithms such as received signal strength (RSS) [15], angle of arrival (AoA) [16], time of arrival (ToA) [17] and time difference of arrival (TDoA) [18] are more widely used. Among above algorithms, RSS offers relatively low positioning accuracy but it can be increased when the algorithm is used in conjunction with the fingerprinting technique in indoor environments [19].

User positioning in indoor VLC environments has been studied extensively. In Ref. [20], an improved version of the existing k-nearest neighbor (kNN) algorithm was proposed. The authors constructed a fingerprinting DB by setting RPs over a 25×25 grid in a 5 m×5 m×3 m environment. Then, in the online step, the RSS between the access point (AP) and the user was measured and the measured value was compared against the fingerprinting DB. Then, the authors proposed a method of adding weights to the kNN algorithm. The revised algorithm reduces the positioning error by assigning large weights to the RPs with high similarity in the kNN algorithm. As a result, the positioning accuracy of the revised kNN algorithm was found to be superior to that of the conventional triangulation method. However, this study did not consider the NLOS scenario in which reflected waves exist.

In Ref. [21], the authors proposed a user-positioning method based on the triangulation method. In this study, the user-positioning results obtained in the LOS scenario and the scenario considering both LOS and NLOS were compared. The results confirmed that when LOS and NLOS were considered together, the positioning error was higher than the case in which only the LOS scenario was considered.

The authors of Ref. [22] proposed two methods to improve the positioning error. The first was related to LED signal selection. Because reflected waves lead to large positioning errors, a method to limit the number of the strongest signals among the received signals for positioning the user was proposed. The second was related to reducing the distance between LEDs. According to this method, the higher the density of LED arrangement, the more uniform is the light intensity distribution throughout the target room and this translates to a higher positioning accuracy. However, when 25 LED APs were deployed in an actual 8 m×8 m×3.5 m indoor environment, the cost was excessive.

In Ref. [23], the authors proposed an optimized LED deployment. The authors considered a scenario with an optimized field of view (FOV). Four, five, nine, and sixteen LEDs were deployed for performance comparison. In this case, the deployment of 5 LEDs is proposed by the authors. The performance evaluation considered received power, illumination, signal to noise ratio (SNR) and root mean square (RMS) delay spread and the proposed method achieved the best performance. This shows that inter-cell interference is minimized while increasing the overall RSS when placing 5 LEDs.

Recently, research using AI technology has been conducted to improve indoor positioning performance. In Ref. [24], the authors proposed a method for positioning based on a fusion of kNN and random forest (RF). The authors achieve precise positioning of 0.069 m at the center, 0.157 m at the edge and 0.166 m at the corner through the proposed method. In addition, the number of trees was optimized to reduce processing time. However, the authors did not consider processing time according to the number of RPs. There is a problem in that the processing time of kNN increases as the number of RPs increases.

The authors of Ref. [25] proposed a method for multiple fingerprinting positioning based on an artificial neural network (ANN). The authors performed multiple localizations simultaneously using an ANN in a real indoor environment. It uses pre-processed RSSI data as input to the ANN. The positioning result achieves an error of 2.68 m when there are 15 targets and verifies that AI can be applied to indoor positioning. However, since only RSSI is used as the input data of ANN, a large error of 2.29 m occurs even when there is only one target.

The key objective of this paper is the joint optimization of positioning error, which is that the processing time is evaluated and the performance is verified by applying AI technology. In particular, when AI is applied, a high-precision positioning is achieved by overcoming the limitations of existing positioning techniques.

Hence, this work studies RSS-based user positioning in indoor VLC environments. Specifically, to achieve precise positioning and reduce processing time, this work proposes a fusion of weighted k-nearest neighbor (WkNN) and deep neural network (DNN). In the existing fingerprinting technique, a large number of RPs is required to achieve high positioning accuracy. However, in real environments, it is difficult to create a large number of RPs and the processing time increases when the positioning algorithm is applied later [26]. The proposed DNN with WkNN significantly reduces the number of RPs required to construct the fingerprinting DB, reduces processing time and increases the positioning accuracy of WkNN by using the DNN model. In addition, in the indoor environment considered in this study, both the LOS and the NLOS scenarios with multipath reflection are considered. The results confirm that the proposed method achieves the highest positioning accuracy.

The main contributions of this paper are as follows:

1. This work considers the LOS and NLOS scenarios together in a VLC environment and proposes a user-positioning method for locating the user by performing AI-based RSS measurements.
2. Under the fingerprinting technique, an AI-based solution is proposed to alleviate the problem of increase in processing time with an increase in the number of RPs. First, after measuring the RSS at the RPs for user positioning, the WkNN component is executed to approximate the user’s location, then, the DNN model is trained using these approximate user location as the input data.
3. The proposed DNN model outputs the user’s final position and the simulation results obtained herein confirm that its positioning accuracy is superior to that of the existing WkNN and triangulation scheme.

The remainder of this paper is organized as follows. Section 2 describes the system model used in this study. Section 3 describes the proposed indoor positioning method. Section 4 discusses the simulation setup and the results obtained in this study. Our concluding remarks are provided in Section 5.

## 2. System Model

This section describes the system model on which the proposed scheme is based. First, we explain the configuration of the indoor VLC environment considered herein. Next, we analyze the channel characteristics of the visible light path in the indoor environment. The visible light path can be divided into a directed path and a non-directed path. Finally, the optical power distribution of the overall channel is shown.

### 2.1. Indoor Environment Configuration

**Figure 1:**
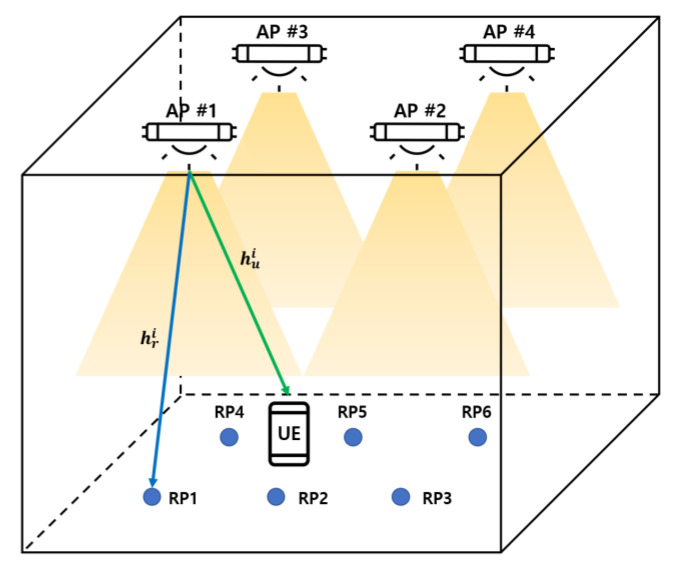
Indoor VLC environment configuration.

The indoor environment used in this study is illustrated in Figure 1. The room size is assumed as 5 m×5 m×3 m and the room is an empty space. LED Aps are uniformly placed on the ceiling at a height of 3 m from the floor. Four LED Aps are used in this model, and each LED has a transmit optical power of 10 W and a half-power half-angle of 60°. Moreover, assume that the receiver moves parallel to the ground at a height of 0.7 m, and the active area of the photo diode (PD) mounted on the receiver is $1{\;\mathrm{cm}}^{2}$. Other parameters of the indoor environment configuration are summarized in Section 4.

### 2.2. Optical Channel Analysis

The optical channel model generally consists of a directed path channel and a non-directed path channel. This model was considered to implement a realistic environment. Therefore, in this study, positioning is performed based on the total optical power received by both channels. For this purpose, the power characteristics of each optical path channel are analyzed to obtain the overall optical power received by the receiver in the indoor environment [24].

**Figure 2:**
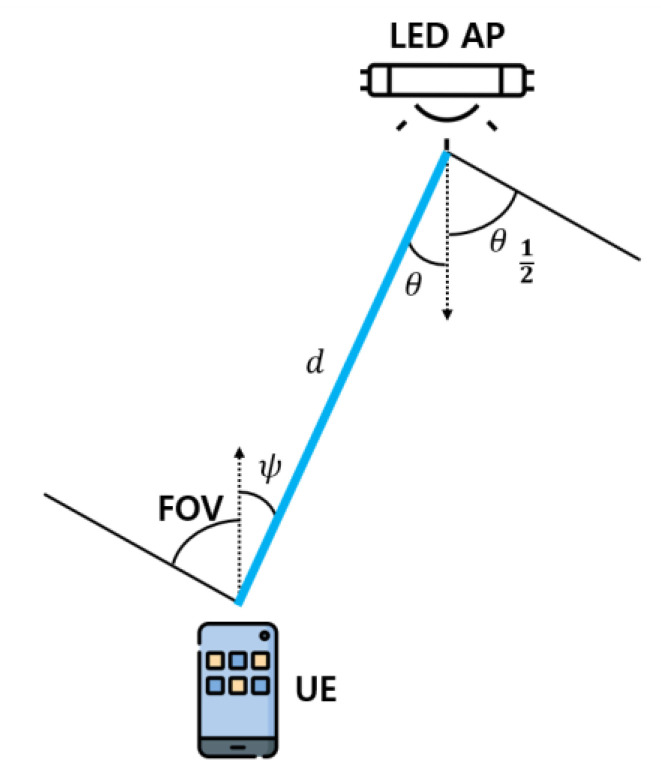
Directed path channel model between LED AP and UE.

First, analyze the directed path optical channel. The directed path between the LED AP and user equipment (UE) is depicted in Figure 2.

In Figure 2, the distance between the LED AP and UE is d, and the received angle is θ. As mentioned earlier, because the UE moves parallel to the ground, the incident angle θ is equal to the irradiation angle of the LED AP. Therefore, the RSS along the directed path of the u-th UE from the i-th LED AP can be obtained using Equation (1) [24].

(1) $$h_{u,\;directed}^{i}=\left(\begin{array}{cc} P_{t}\frac{A\left(m+1\right)}{2\pi d^{2}}\cos^{m}\left(\theta \right)T_{s}\left(\psi \right)C\left(\psi \right)\cos\left(\psi \right), & 0\leq \psi \leq \psi_{c}\\ 0 & \psi_{c}<\psi \end{array}\right)$$

where $P_{t}$ is the transmission optical power of the LED AP, m is the Lambertian order, A is the active area of the UE, d is the distance between the LED AP and UE, $T_{s}\left(\psi \right)$ is the optical filter gain, and C(ψ) is the optical concentration gain. In addition, $\psi_{c}$ denotes the FOV of the UE.

**Figure 3:**
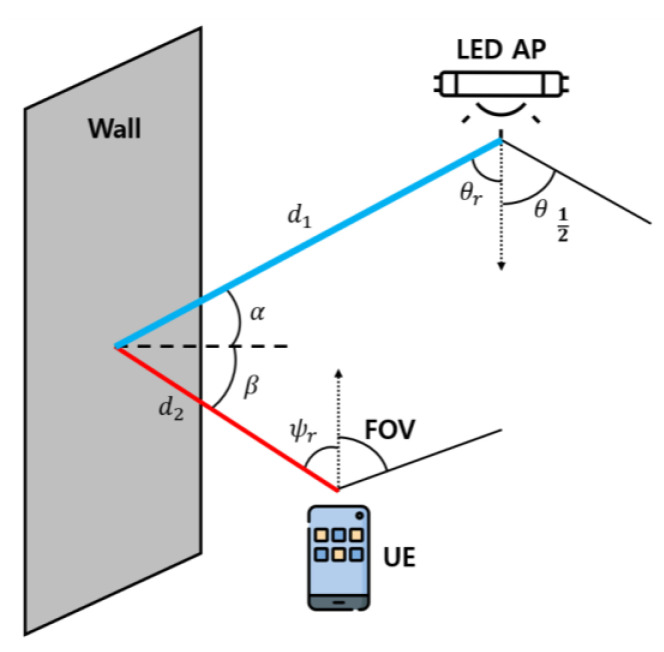
Non-directed path channel model between LED AP and UE.

Next, we analyze the non-directed path optical channel. The non-directed path between the LED AP and UE is depicted in Figure 3.

**Figure 4:**
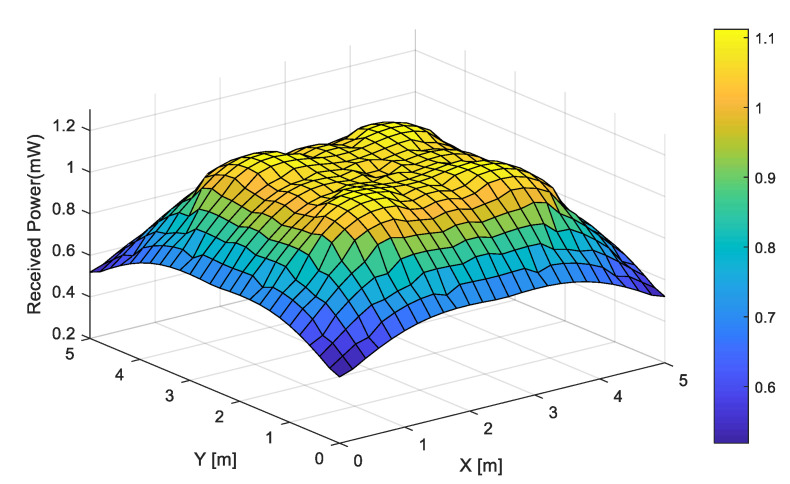
RSS distribution of overall channel.

As can be seen in Figure 3, the non-directed path considers the first reflected wave. In this case, the distance from the LED AP to the reflection point of wall is $d_{1}$, incident angle is α, distance from the reflection point to the UE is $d_{2}$, and reflection angle is β. In addition, $\theta_{r}$ is the irradiation angle between the LED AP and the wall, and $\psi_{r}$ is the angle at which the UE receives optical power from the wall. Accordingly, the RSS from the i-th LED AP through the non-directed path of the u-th UE can be obtained using Equation (2) [24].

(2) $$h_{u,\;non-directed}^{i}=P_{t}\frac{A\left(m+1\right)}{2\pi d_{1}^{2}d_{2}^{2}}\rho \cos^{m}\left(\theta \right)dA_{wall}\cos\left(\alpha \right)\cos\left(\beta \right)T_{s}\left(\psi_{r}\right)C\left(\psi_{r}\right)\cos\left(\psi_{r}\right)$$

where $dA_{wall}$ is the surface element of the wall, and ρ is the reflection coefficient of the wall. Furthermore, the range of $\psi_{r}$ is $0\leq \psi_{r}\leq \psi_{c}$. Then, according to (1) and (2), the overall RSS of the UE can be expressed using Equation (3) [24], and the corresponding RSS distribution is depicted in Figure 4.

(3) $$h_{overall}^{\;}=h_{directed}^{\;}+h_{non-directed}^{\;}$$

## 3. Proposed Positioning Scheme

In this section, the proposed positioning technique is explained. This technique uses fingerprinting, WkNN, and DNN. As mentioned in the Section 1, the fingerprinting technique and WkNN are used in the existing indoor positioning scheme. In the existing method, a large number of RPs are required to achieve high positioning accuracy. However, the positioning processing time of WkNN increases as the number of RPs increases. In addition, there are maintenance-related difficulties with changes in the actual indoor environment.

**Figure 5:**
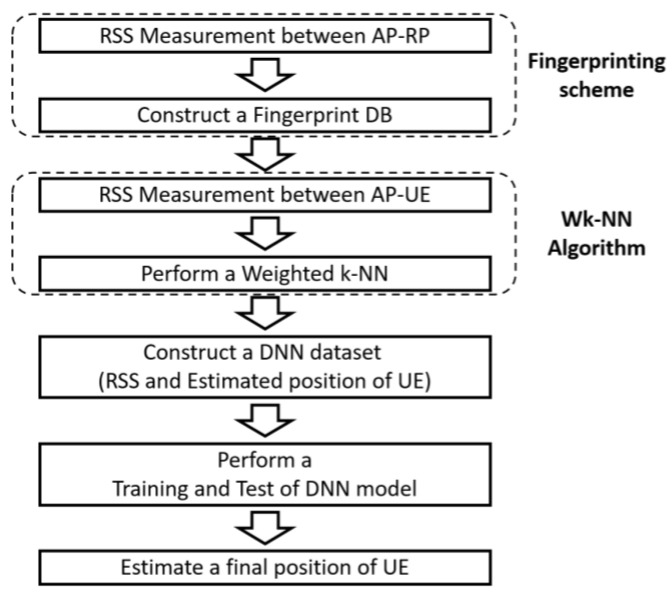
Block diagram of proposed system.

Therefore, this study aims to develop a positioning system that is characterized by fast processing time and precise positioning by reducing the number of RPs. To this end, we apply DNN, an AI technology. The core of the proposed method is using the approximate coordinates of the UE obtained through fingerprinting and WkNN as the inputs to the DNN model. The system flow is shown in Figure 5. The sequence of parameter determination is described in the subsections of this section.

### 3.1. Fingerprinting Method

Fingerprinting is a common method for indoor positioning and it can be used in conjunction with RSS to achieve a higher positioning accuracy than that of the conventional techniques. The fingerprinting technique requires a DB that stores the RSS information of the entire map on which positioning is to be performed. Therefore, the fingerprinting technique consists of two steps. First, the offline stage involves collecting RSS data based on an RP. The RP is represented by specific coordinates in the entire map, and the RSS is measured for each AP. The greater the number of RPs, the higher is the positioning accuracy. However, when the number of RPs increases in an actual indoor environment, the time required for DB construction and maintenance increase. In addition, the processing time for indoor positioning increases. Therefore, when initially determining the number of RPs, two aspects should be considered, namely positioning accuracy and total processing time.

**Figure 6:**
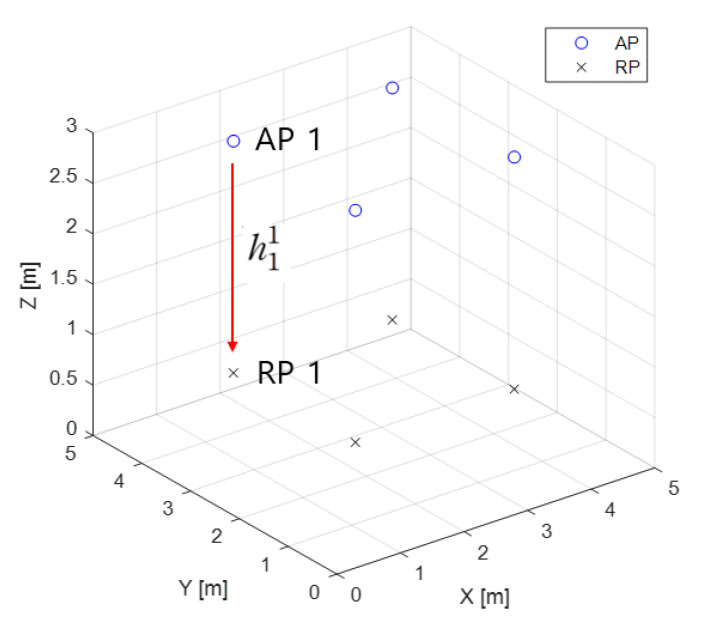
Concept of fingerprinting DB measurements.

Figure 6 shows the concept of measuring the RSS at RPs to construct a fingerprinting DB. In this study, the number of RPs is set to 16. The rationale for this choice is detailed later in the Section 4. The RSS measured at each RP is stored in the fingerprinting DB, which can be expressed as follows.

(4) $$H_{DB}=\left[\begin{array}{ccccc} h_{1}^{1} & \cdots & h_{1}^{i} & \cdots & h_{1}^{I}\\ \vdots & & \vdots & & \vdots \\ h_{r}^{1} & \cdots & h_{r}^{I} & \cdots & h_{r}^{i}\\ \vdots & & \vdots & & \vdots \\ h_{R}^{1} & \cdots & h_{R}^{i} & \cdots & h_{R}^{I} \end{array}\right]$$

where $h_{r}^{i}$ denotes the RSS between the i-th AP and the r-th RP. The constructed fingerprinting DB is later used to determine the approximate user position by means of WkNN.

### 3.2. Weighted k-Nearest Neighbor (WkNN)

kNN is a machine learning method used to determine proximity through feature data comparison of each object. The WkNN method assigns higher weight to the data with high proximity based on the proximity derived through kNN. To apply this method to indoor positioning, proximity is estimated using the available RSS information. Therefore, the proximity between the RSS of each RP and the RSS of the actual UE is obtained from the DB constructed using the fingerprinting method. In kNN, proximity can be obtained using two methods, namely the Euclidean distance method and the Manhattan distance method. Herein, we employ the Euclidean distance method, which is generally used for positioning. WKNN is performed as follows [20]. First, the RSS of the UE is expressed as follows.

(5) $$H_{u}^{\;}=(h_{u}^{1},\;h_{u}^{2},\;\ldots ,\;h_{u}^{I})$$

where $h_{u}^{i}$ represents the RSS value between i-th AP and the u-th UE. The Euclidean distance $d_{u,\;r}$ can then be computed as follows (Equation (6)) [20].

(6) $$d_{u,\;r}=\sqrt{\sum_{i=1}^{I}{\left(h_{u}^{i}-h_{r}^{i}\right)}^{2}}$$

Then, the WkNN weight can be determined from the computed Euclidean distance. The smaller the computed Euclidean distance, the higher is the weight, and vice versa. The weight can be obtained using Equation (7) [20].

(7) $$w_{u,\;r}=1-\frac{d_{u,\;r}}{\sum_{r=1}^{R}d_{u,\;r}}$$

After obtaining the weights using Equation (7), the approximate UE location can be determined. The three-dimensional coordinates of the UE may be obtained using Equation (8) [20].

(8) $$X_{e}=\frac{\sum_{r=1}^{R}w_{u,\;r}x_{r}}{\sum_{r=1}^{R}w_{u,\;r}},\;Y_{e}=\frac{\sum_{r=1}^{R}w_{u,\;r}y_{r}}{\sum_{r=1}^{R}w_{u,\;r}},\;Z_{e}=\frac{\sum_{r=1}^{R}w_{u,\;r}z_{r}}{\sum_{r=1}^{R}w_{u,\;r}}$$

**Figure 7:**
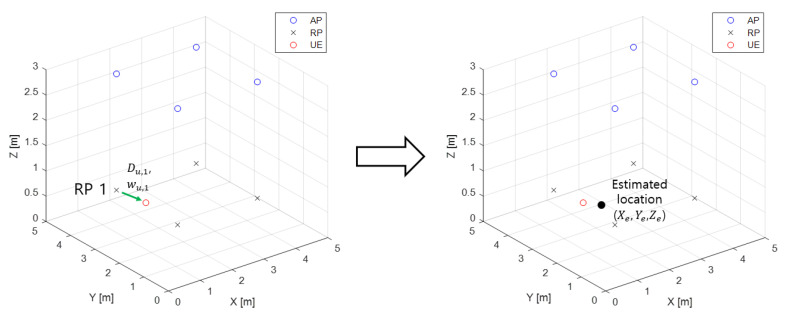
Processing step of WkNN.

Figure 7 illustrates the processing step of WkNN. When the approximate coordinate of the UE are estimated following the aforementioned process, the execution of WkNN is completed and an input datum for training the DNN model is obtained.

### 3.3. Deep Neural Network (DNN)

DNN is an AI method that can be applied in various fields to a given input/output dataset. First, to train the DNN model, a dataset is constructed with the correct answers. In this study, the data for training the DNN model are constructed to perform indoor positioning. Hence, the trained DNN model outputs the three-dimensional coordinates of the UE. In the constructed training dataset, the input and output are expressed using Equations (8) and (9), respectively.

(9) $$Input\;Data=\left(h_{u}^{i},\;X_{e},\;Y_{e},Z_{e}\right)\;\;\left(where,\;i=1,2,\ldots I.\right)$$

(10) Output Data=(X, Y, Z)

where i denotes the number of the APs transmitting the signal. As the input data for the DNN model, we employ the approximate positioning results between the AP and the UE, as obtained using RSS and WkNN. The output data of the DNN model are the final estimated coordinates of the UE. Table 1 presents the structure of the DNN model used in this study. As can be inferred from Table 1, the dropout technique was used in consideration of the generalization performance of the DNN model. The designed DNN model uses the Adam optimizer.

The proposed DNN model is optimized in terms of two parameters, namely the number of layers and the learning rate. The performance of the optimized DNN model is as follows: training accuracy of the model = 95.57%, training loss = 0.0021, test accuracy = 99.37%, and testing loss = 0.00024. The training and test results indicate that the training and generalization performance of the proposed DNN model are reliable.

### 3.4. Parameter Determination

This subsection describes how to determine the number of RPs for indoor positioning, in addition to the parameters related to the design of the DNN model. First, the number of RPs was set through comparison with the triangulation method. The WkNN and kNN methods are dependent on the number of RPs. Therefore, as the number of RPs increases, the positioning accuracy increases. In Table 2, the positioning processing time and error are compared as the number of RPs is increased from 4 to 9 and 16. The comparison showed that WkNN offered advantages over the triangulation method in terms of processing time and positioning error when the number of RPs was 16. Therefore, the number of RPs was set to 16 in the proposed scheme.

After determining the number of RPs, we optimized the hyperparameters of the DNN model. Among the hyperparameters, we optimized the number of layers and the learning rate. In Table 3, the performance of the DNN model for various numbers of layers is summarized. Here, the number of layers refers to the total number of layers, including inputs and outputs. According to Table 3, the four-layer structure achieved the best performance with an accuracy of 99.37% and a loss of 0.00024. In this case, the numbers of nodes in 1–4 layers of the four-layer structure were 7, 210, 50 and 3, respectively. The learning rate was fixed to 0.005.

Next, the performance of the DNN model was tested in various learning rates and the results are summarized in Table 4. The learning rates were varied from 0.01 to 0.005 and 0.001. In this case, the DNN model of the four-layer structure was used based on the results summarized in Table 3. As presented in Table 4, when the learning rate was set to 0.005, the best test accuracy and positioning error were achieved.

Hence, in this study, we set the number of RPs to 16 based on the aforementioned results and optimized the DNN model in terms of the number of layers and learning rate. The other simulation parameters are described in the following sections.

## 4. Simulation and Evaluations

In this section, the simulation parameter and results are presented and discussed. In Section 4.2, the performance of the proposed scheme is compared with that of the existing scheme.

### 4.1. Simulation Parameters

This section describes the parameters used in the simulation. The parameters themselves are listed in Table 5. The indoor environment was assumed as an empty space measuring 5 m × 5 m × 3 m. In total, four APs were used in the indoor environment and a total of 16 RPs were used, as summarized in Table 2. A total of 80,000 training datasets and 20,000 test datasets were used to train and test the DNN model, respectively. The simulation was repeated for 10,000 iterations, it was coded using MATLAB 2017b and Python 3.7.

### 4.2. Simulation Results and Discussion

**Figure 8:**
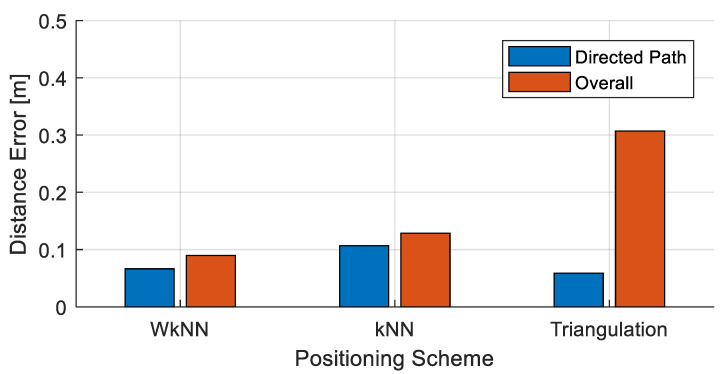
Comparison of positioning errors of each scheme.

In this section, the simulation results are presented and discussed. The simulation results are presented in terms of positioning accuracy and processing time. First, the positioning accuracy is computed using the DNN, which is depicted in Figure 8. Figure 8 shows the results obtained only for the directed path channel and those obtained for the overall channel. For the directed path channels, the triangulation technique yielded the lowest positioning error. Thus, the triangulation method can perform more accurate positioning than the WkNN and kNN in the directed path channel. In the overall channel, the positioning errors of the three techniques increased. In this case, the positioning errors of the WkNN and kNN algorithms, which perform fingerprinting-based positioning, were smaller than those of the triangulation method. According to these results, the positioning results obtained using these methods were more stable than those obtained using the triangulation method because these methods performed positioning using the fingerprinting method. Among these methods, the WkNN method yielded the lower positioning error of 0.0898 m. Hence, the approximate positioning results, which represent the input to the DNN model, influence the final positioning results of the DNN model.

Next, the processing time is analyzed for the positioning. Herein, we proposed a method to train the DNN model by using the approximate positioning results obtained using WkNN. The processing time required to achieve the positioning error of 0.0898 m can be determined as follows. First, as summarized in Table 2, the execution time of the WkNN algorithm in an environment with 16 RPs is 0.00048 s. Then, when the approximate positioning results obtained using WkNN are input into the trained DNN model, the model requires 1–2 μs to generate an output. Hence, since the processing time of the DNN model is extremely short, the overall processing time is not affected significantly. Meanwhile, the DNN model improves the positioning accuracy and facilitates more precise positioning.

## 5. Conclusions

This paper presents a system that applies DNN based AI technology for accurate positioning in an indoor VLC environment. In particular, this work aims to improve the indoor positioning accuracy by optimizing the input data of the DNN model. First, it calculates the RSS between the AP and UE by analyzing the VLC environment. The obtained RSS is used as input data of the DNN model. Then, it obtains the training data of DNNs by using the fingerprinting techniques and WkNN methods, which are generally used for indoor positioning. Hence, the various data can be included for training the DNN model. The more the data, the better is the training and testing performance of the DNN model. The DNN model can be further improved through the optimization of hyperparameters. The proposed DNN model optimized the number of layers and learning rates. Hence, the proposed scheme attained an average positioning error of 0.0898 m with a processing time of 0.5 ms. The simulation results show that the positioning error was reduced and a lower processing time achieved in an indoor environment. Further work can include building a testbed in order to verify the performance of the proposed method in a real environment.

## Figures and Tables

**Table 1:** Detailed structure of DNN model.

Layer Name	Number of Nodes, Activation
Input Layer	7
Hidden Layer 1	210, ReLU
Drop out 1	0.4
Hidden Layer 2	50, ReLU
Drop out 2	0.4
Output Layer	3, Sigmoid

**Table 2:** Comparison between proposed and conventional scheme.

Performance	Number of RPs	WkNN [20]	kNN [27]	Triangulation [21]
Processing time [s]	4	0.00021	0.00019	0.00237
	9	0.00033	0.00026	
	16	0.00048	0.00039	
Positioning error [m]	4	1.687	1.711	1.298
	9	1.324	1.351	
	16	0.812	0.846	

**Table 3:** Performance analysis for various numbers of layers.

Number of Layers	Training/Test Loss	Training/Test Accuracy	Positioning Error [m]
7-Layer	0.234/0.0046	82.07/91.82	0.4104
6-Layer	0.0049/0.00085	93.38/98.32	0.1746
5-Layer	0.0023/0.00025	95.04/99.30	0.0913
4-Layer	0.0021/0.00024	95.57/99.37	0.0898
3-Layer	0.0011/0.0003	97.26/98.4	0.0942

**Table 4:** Performance analysis for various learning rates.

Learning Rate	Training/Test Loss	Training/Test Accuracy	Positioning Error [m]
0.01	0.0021/0.00025	95.40/99.14	0.0933
0.005	0.0021/0.00024	95.57/99.37	0.0898
0.001	0.0021/0.00026	95.54/98.65	0.0961

**Table 5:** Simulation parameters.

Parameter		Value
Environment	Room size	5 m × 5 m × 3 m
	Number of APs	4
	Number of RPs	16
	Reflection coefficient	0.8
Transmitters	Transmit power	10 W
	Half power semi-angle	60°
	Wavelength	420 nm
	Elevation	−90°
Receiver	Active area of UE	$1{\;\mathrm{cm}}^{2}$
	Field of view (FOV)	60°
	Optical filter gain	1
	Optical concentrator gain	1.5

## Data Availability

Not applicable.

## References

[1] Deng Y., Ai H., Deng Z., Gao W., Shang J. (2022). An Overview of Indoor Positioning and Mapping Technology Standards. Standards.

[2] Kumari A., Bhatt D. (2022). Advanced System Analysis and Survey on the GPS Receiver Front End. IEEE Access.

[3] Sadowski S., Spachos P. (2018). RSSI-based indoor localization with the Internet of Things. IEEE Access.

[4] Basiri A., Lohan E.S., Moore T., Winstanley A., Peltola P., Hill C., Amirian P., Silva P.F. (2017). Indoor location based services challenges, requirements and usability of current solutions. Comput. Sci. Rev..

[5] Li N., Chen J., Yuan Y. (2016). A WiFi indoor localization strategy using particle swarm optimization based artificial neural networks. Int. J. Distrib. Sens. Netw..

[6] Farahsari P.S., Farahzadi A., Rezazadeh J., Bagheri A. (2022). A Survey on Indoor Positioning Systems for IoT-Based Applications. IEEE Int. Thin. J..

[7] Shang S., Wang L. (2022). Overview of WiFi fingerprinting-based indoor positioning. IET Commun..

[8] Alarifi A., Al-Salman A., Alsaleh M., Alnafessah A., Al-Hadhrami S., Al-Ammar M.A., Al-Khalifa H.S. (2016). Ultra wideband indoor positioning technologies: Analysis and recent advances. Sensors.

[9] Wang Q., Feng Y., Zhang X., Sun Y., Lu X. (2016). IWKNN: An effective Bluetooth positioning method based on Isomap and WKNN. Mob. Inf. Syst..

[10] Zhuang Y., Hua L., Qi L., Yang J., Cao P., Cao Y., Wu Y., Thompson J., Haas H. (2018). A Survey of Positioning Systems Using Visible LED Lights. IEEE Commun. Surv. Tutor..

[11] Lian J., Vatansever Z., Noshad M., Brandt-Pearce M. (2019). Indoor visible light communications, networking, and applications. J. Phys. Photonics.

[12] Zhu Y., Chen X. Visible Light Communication System Based on White LED. Proceedings of the IEEE International Conference on Artificial Intelligence and Computer Applications (ICAICA).

[13] Priya C.B., Sivakumar S. (2018). A survey on localization techniques in wireless sensor networks. Int. J. Engi. Technol..

[14] Ullah I., Chen J., Su X., Esposito C., Choi C. (2019). Localization and Detection of Targets in Underwater Wireless Sensor Using Distance and Angle Based Algorithms. IEEE Access.

[15] Guo X., Shao S., Ansari N. (2017). Khreishah, A. Indoor Localization Using Visible Light via Fusion of Multiple Classifiers. IEEE Photonics J..

[16] Eroglu Y.S., Guvenc I., Pala N., Yuksel M. AOA-based localization and tracking in multi-element VLC systems. Proceedings of the IEEE 16th Annual Wireless and Microwave Technology Conference (WAMICON).

[17] Krishnaveni B.V., Reddy K.S., Reddy P.R. An Introduction to the TOA measurement for UWB indoor localization Systems. Proceedings of the 5th Conference on Information and Communication Technology (CICT).

[18] Han M., Zeng G. Research on indoor radio frequency positioning algorithm based on TDOA. Proceedings of the International Seminar on Computer Science and Engineering Technology (SCSET).

[19] Zhao C., Zhang H., Song J. Fingerprint and visible light communication based indoor positioning method. Proceedings of the 9th International Conference on Advanced Infocomm Technology (ICAIT).

[20] Van M.T., Tuan N.V., Son T.T., Le-Minh H., Burton A. (2017). Weighted k-nearest neighbor model for indoor VLC positioning. IET Commun..

[21] Mohammed N.A., Elkarim M.A. (2015). Exploring the effect of diffuse reflection on indoor localization systems based on RSSI-VLC. Opt. Express.

[22] Gu W., Aminikashani M., Deng P., Kavehrad M. (2016). Impact of Multipath Reflections on the Performance of Indoor Visible Light Positioning Systems. J. Light. Technol..

[23] Gismalla M.S.M., Abdullah M.F.L., Ahmed M.S., Mabrouk W.A., Fadhali N.A., Saeid E., Supa’at A., Das B. (2021). Design and Analysis of Different Optical Attocells Deployment Models for Indoor Visible Light Communication System. Int. J. Integ. Eng..

[24] Tran H.Q., Ha C. (2019). Fingerprint-Based Indoor Positioning System Using Visible Light Communication—A Novel Method for Multipath Reflections. Electronics.

[25] Yoo J. (2022). Multiple Fingerprinting Localization by an Artificial Neural Network. Sensors.

[26] Xia S., Liu Y., Yuan G., Zhu M., Wang Z. (2017). Indoor Fingerprint Positioning Based on Wi-Fi: An Overview. Int. J. Geo-Inf..

[27] Ge X., Qu Z. Optimization WIFI indoor positioning KNN algorithm location-based fingerprint. In Proceeding of the 7th IEEE International Conference on Software Engineering and Service Science (ICSESS).

